# Synthesis of Polyaniline Coating on the Modified Fiber Ball and Application for Cr(VI) Removal

**DOI:** 10.1186/s11671-021-03509-y

**Published:** 2021-04-08

**Authors:** Xiao Li Ma, Guang Tao Fei, Shao Hui Xu

**Affiliations:** 1 Key Laboratory of Materials Physics and Anhui Key Laboratory of Nanomaterials and Nanotechnology, Institute of Solid State Physics, Hefei Institutes of Physical Science, Chinese Academy of Sciences, P. O. Box 1129, Hefei, 230031 People’s Republic of China; 2 University of Science and Technology of China, Hefei, 230026 People’s Republic of China

**Keywords:** Polyaniline, Fiber ball, Chromium, Adsorption, Regeneration

## Abstract

**Abstract:**

In this study, polyaniline (PANI) is prepared by means of chemical oxidization polymerization and directly loaded on the modified fiber ball (m-FB) to obtain macroscale polyaniline/modified fiber ball (PANI/m-FB) composite, and then its removal ability of Cr(VI) is investigated. The effects of different parameters such as contact time, pH value and initial concentration on Cr(VI) removal efficiency are discussed. The experimental results illustrate that the favorable pH value is 5.0 and the maximum removal capacity is measured to be 293.13 mg g^−1^. Besides, PANI/m-FB composites can be regenerated and reused after being treated with strong acid. The kinetic study indicates that the adsorption procedure is mainly controlled by chemical adsorption. More importantly, the macroscale of composites can avoid secondary pollution efficiently. Benefiting from the low cost, easy preparation in large scale, environmentally friendly, excellent recycling performance as well as high removal ability, PANI/m-FB composites exhibit a potential possibility to remove Cr(VI) from industrial waste water.

**Graphic Abstract:**

The polyaniline (PANI) was coated on modified fiber ball (m-FB) to remove Cr(VI) in waste water, and this kind of PANI/m-FB composites can avoid secondary pollution efficiently due to its macrostructure. Furthermore, the removal capacity can reach to 291.13 mg/g and can be multiple reused.
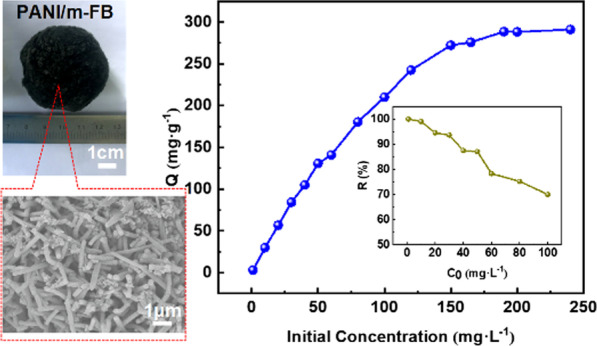

**Supplementary Information:**

The online version contains supplementary material available at 10.1186/s11671-021-03509-y.

## Background

With the rapid development of industry, the environmental contamination has been more and more serious, and the pollution caused by heavy metal ions is one of the most severe challenges that humans have to conquer with [1]. Particularly, hexavalent chromium [Cr(VI)], which resulted from electroplating, textile printing and mordanting, can generate great damage to the environment and even human health due to its high toxicity, carcinogenic effects, easy mobility, and ability of accumulation in ecosystem and human body [2–4]. Compared with Cr(VI), the toxicity of Cr(III) is much less than that of Cr(VI) and easier to be removed by adsorption and precipitation [3]. Hence, the deoxidation of Cr(VI) to less poisonous Cr(III) and then adsorption and precipitation prior to its discharge to the environment are essential to ensure the protection of aquatic lives and human in current researches [5–9].

It is reported that polyaniline (PANI) has a prominent ability that can reduce Cr(VI) to Cr(III) due to its distinct oxidation characteristic, higher reaction rate and better stability [10]. Besides, PANI contains plentiful positively charged amine and imine groups which can be utilized as a promising adsorption material to adsorb the Cr(III) as the reduction product of Cr(VI). So, there has been a great deal of research toward the use of PANI for Cr(VI) removal due to its easy synthesis, low cost, remarkable environmental stability and reversibility [11–13]. Up to now, people have fabricated various morphologies of polyaniline like polyaniline films, polyaniline nanowires, polyaniline-based composites and so on [11, 14–18], whereas there are still many problems desiring to be solved. For one thing, the specific surface areas of PANI films are relatively small resulting in a decreasing contact with Cr(VI) solution and limiting the removal capacity [19]. For another, compared with the films, although the removal capacities of PANI nanowires and polyaniline-based composites have enhanced enormously due to the large specific surface area, the sizes of these materials are too small to be recycled totally and it can cause secondary pollution in industrialized application. Hence, how to effectively solve the problem of secondary pollution while improving the removal capacity in order to make it widely used in industrial wastewater treatment rather than just in laboratory research is still a great challenge. Up to now, however, far too little attention has been paid to this aspect.

Fiber ball (FB), consisting of polyester or polyacrylonitrile fiber, is a kind of sphere structure caused by the curving of long fibers through the method of fabricating non-woven fabrics. As a burgeoning technology in water treatment, fiber ball has been widely applied to industrial waste water treatment, drinking water treatment and seawater treatment due to its low cost, good elasticity, stable physical and chemical properties, strong pollutant interception ability and fast filter speed [20–22]. More importantly, this kind of macroscale fiber ball will not generate secondary pollution at all so that if we load PANI on the surface of fiber ball to obtain polyaniline/fiber ball (PANI/FB) composites, the problem of secondary pollution caused by nanoscale PANI will be solved.

The aim of this study is to solve secondary pollution caused by PANI at nanoscale during the process of Cr(VI) treatment and realize the regeneration and recycle of adsorbents. Herein, PANI is prepared and directly loaded on the macroscale modified fiber ball (m-FB) to obtain the PANI/m-FB composite. The experimental results show that PANI is firmly combined with fiber balls and the PANI/m-FB composite not only exhibits an effective removal capacity of Cr(VI) in aqueous solution, but also can be regenerated and reused. Hence, it is more beneficial to the extension of industrialization considering the fact of its easy synthesis, remarkable environmental stability and reversibility [23, 24].

## Methods

### Materials

Aniline, ammonium persulfate (APS), tartaric acid (TA), sulfuric acid (H_2_SO_4_), hydrogen peroxide (H_2_O_2_) and potassium dichromate (K_2_Cr_2_O_7_) were all purchased from Aladdin biochemical technology Co., LTD., Shanghai, China. All the reagents were analytically pure and were used without further purification. Fiber balls (FB) which mainly composed of polyester fibers were obtained commercially from Yijia water purification material Co., LTD., in Henan, China. All the solutions were prepared by deionized water.

### Preparation of Modified Fiber Ball (m-FB)

Firstly, fiber balls were soaked into deionized water for 1 h at room temperature and cleaned in ultrasonic washing units for 20 min three times to remove the dust or impurities and then dried at 60 °C in drying oven for 12 h. Subsequently, fiber balls were immerged in 10 g L^−1^ H_2_O_2_ solution and stirred for 24 h at room temperature in order to modify the surface of fiber balls. And then modified fiber balls were rinsed by deionized water three times again and then dried at 60 °C in drying oven for 12 h.

### Preparation of PANI/m-FB Composites

Aniline (4 mL) and tartaric acid (0.1 mol) were dissolved in 100 ml deionized water at room temperature with magnetic stirring for 15 min, and APS (0.4 mol) was dispersed in 100 ml deionized water as well. After placing the aniline solution as well as APS solution in an ice bath for 5 min, respectively, APS solution was poured into aniline solution slowly and then put the modified fiber ball into the mixed solution [11]. Next, the reaction mixture was put in an ice bath and then the chemical oxidative polymerization took place. After 24 h, the products were purified by filtering and rinsing with deionized water and alcohol for several times to remove the excess acid and by-products. Finally, the resulting composites were dried at 60 °C in drying oven for 12 h.

For comparison, we prepared PANI/FB composites and the preparation procedure was similar to that of PANI/m-FB composites except for the modification of fiber ball.

### Characterization of PANI/m-FB Composites

The morphology of PANI/m-FB was characterized by field emission scanning electron microscope (FESEM, Horiba Company in Japan, SU8020). The molecular structure and functional groups were characterized by the Fourier transform infrared spectroscopy (FT-IR, JASCO FT-IR 410) in the range of 500 to 4000 cm^−1^ at a resolution of 4 cm^−1^. The optical absorbance of Cr(VI) solution was tested by ultraviolet–visible spectrophotometer (UV–Vis, UV1750), and the oxidation state of chromium adsorbed on composites was analyzed by X-ray photoelectron spectra (XPS, Thermo ESLCALAB 250Xi).

## Results and Discussion

**Fig. 1:**
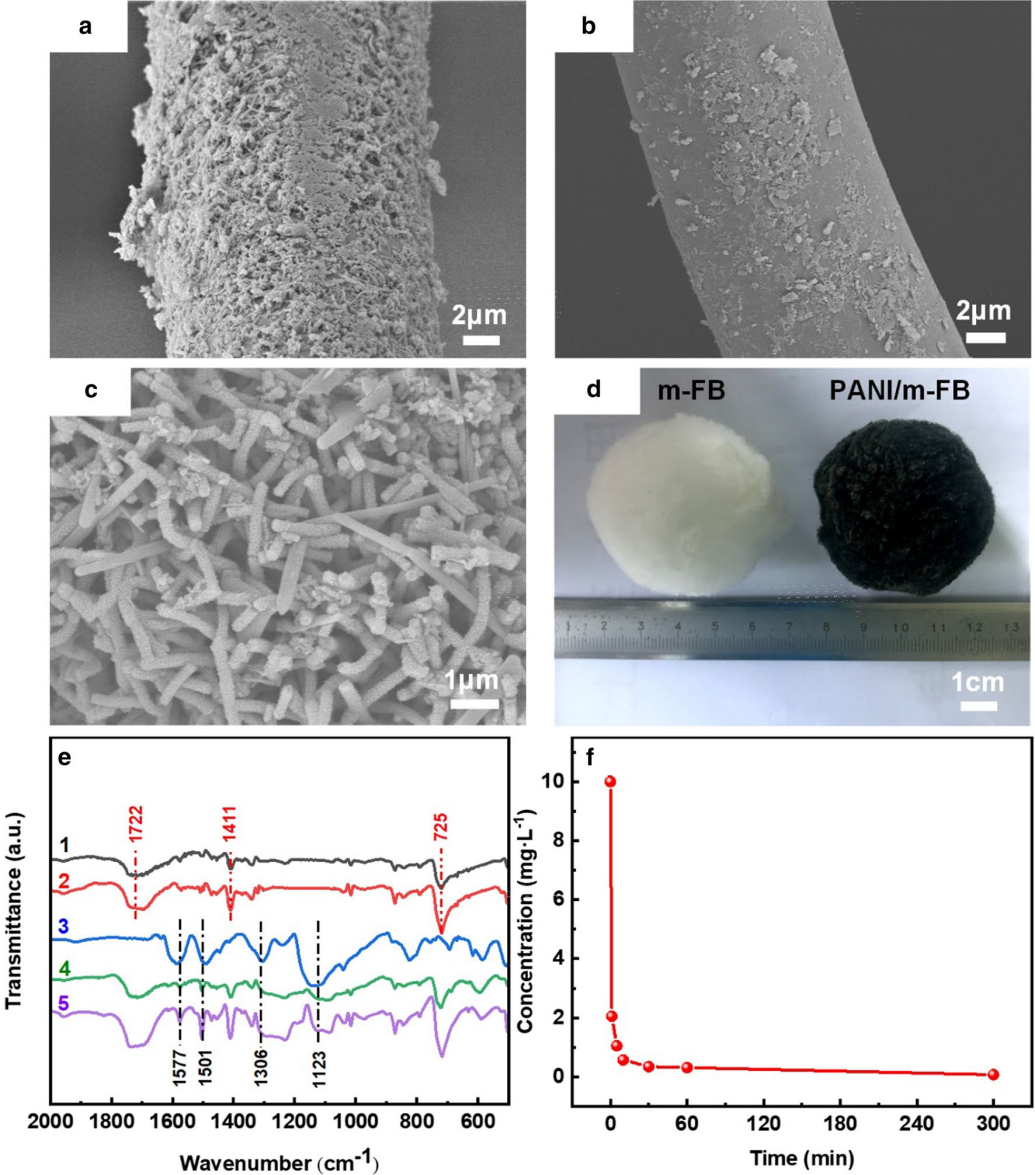
**a** SEM image of PANI/m-FB; **b** SEM image of PANI/FB; **c** enlarged image of (**a**); **d** the digital image of fiber ball and PANI/m-FB composite, respectively; **e** FT-IR spectra of (1) FB; (2) m-FB; (3) PANI powder; (4) PANI/FB; (5) PANI/m-FB; **f** changes of remanent Cr(VI) concentration at different times (*T* = 303 K, *C*_0_ = 10 mg L^−1^, pH = 5.0)

Figure 1a–d exhibits the SEM images and the digital camera photograph of the samples, respectively. Figure 1a, b illustrates the microstructure of PANI/m-FB and PANI/ FB composites, respectively, and it can be noticed that PANI coated on the modified fiber ball was more homogeneous, indicating that modification can enhance the ability of fiber ball to load PANI. Furtherly, Fig. 1c is the enlarged picture of Fig. 1a, and from Fig. 1c, it can be seen that the morphology of PANI is mainly composed of one-dimensional nanostructure with the diameter of about 190 ± 10 nm. Besides, Fig. 1d shows the macrostructure image of fiber ball before and after coated by PANI.

To investigate the load capacity of fiber ball, we chose three different groups of modified fiber balls and weighed their weights. Then, we loaded PANI on them under the same condition to obtain PANI/m-FB composites and numbered them as Sample-1 to Sample-3. For comparison, we measured the load rate of PANI/FB composites and marked as Sample-4 in the same way. After cleaned by deionized water in ultrasonic washing units for 10 min several times, the weight was measured and the results are shown in Table 1. It can be concluded that the average PANI load rate of modified fiber ball is about 5.65%, which is much superior to the fiber ball without modification. So, we selected modified fiber balls to load PANI in the subsequent experiments.

**Table 1:** The PANI load rate of fiber balls

	Sample-1	Sample-2	Sample-3	Sample-4
*m*_0_ (g)	4.3030	3.5680	3.6771	3.7177
*m*_1_ (g)	4.5565	3.7725	3.8735	3.8214
*η*_1_ (%)	5.89	5.73	5.34	2.79
Average *η*_1_ (%)	5.65			/

*m*_0_ represents the mass of modified and unmodified fiber ball, *m*_1_ represents the mass of samples after cleaned by deionized water in ultrasonic washing units for several times, *η*_1_ represents the load rate of PANI

Figure 1e exhibits the FT-IR spectra of different samples. Curves 1 and 2 reveal the spectrum of fiber ball and modified fiber ball, respectively. The adsorption peaks appeared at Curves 1 and 2 located at 1722 cm^−1^, 1411 cm^−1^ and 725 cm^−1^ are corresponding to C=O stretching vibrations, C–O stretching vibrations of carboxylic acids and OH out-of-plane bending vibration, respectively [25]. Compared with Curve 1, the adsorption peaks in Curve 2 are enhanced apparently, which may arise from the formation of carboxyl and hydroxyl radicals in the fiber balls under the reaction of strong oxidants [26–28]. In a word, it indicates that the physical and chemical bonding forces of the fibers are enhanced and the surface atoms are more active after being modified with 10 g L^−1^ H_2_O_2_. In Fig. 1e, Curve 3 is the FT-IR spectrum of PANI powder and the characteristic peaks located at 1577 cm^−1^ and 1501 cm^−1^ are corresponding to the stretching vibrations of C=C bond on reduction units benzene structure (NH–B–NH) and oxidation units quinone structure (N=Q=N), where Q, B represent quinone ring and benzene ring, respectively [11, 29]. Both the reduction units (NH–B–NH) and oxidation units (N=Q=N) appearing in the PANI mean that the PANI we fabricated can be further oxidized or reduced. The peak at 1306 cm^−1^ is related to the C–N bond stretching vibration on the benzene ring, and the peak at 1123 cm^−1^ is due to the characteristic absorption of C-H vibration in B-NH^+^ = Q [29, 30]. Curves 4 and 5 in Fig. 1e exhibit the FT-IR spectrum of PANI/FB and PANI/m-FB composites, respectively, and it can be obviously seen that the peaks related to the groups of PANI also appeared in the FT-IR of composites.

In our experiment, we adopt the method of standard concentration curve to label the concentration of Cr(VI) in aqueous solution.

Additional file 1: Fig. S1(a) is the optical absorption curves for different Cr(VI) concentrations, and it can be noticed that as the Cr(VI) concentration increases, the optical absorption enhances as well. In terms of the relation between peak values at 350 nm with the Cr(VI) concentration in Additional file 1: Fig. S1(a), a linear fitting curve is plotted in Additional file 1: Fig. S1(b) and the equation can be derived as follows:

1 $$Y = 0.02702X + 0.06551$$

where *Y* and *X* represent the optical absorbance and concentration of Cr(VI) in aqueous solution, respectively.

For the removal and adsorption experiment, 1.0 g PANI/m-FB composites were put into 150 mL Cr(VI) solution with the initial concentration (*C*_0_) of 10 mg L^−1^, pH = 5.0. And then, 3 mL of the reaction solution was taken out for optical absorption measurement at predetermined intervals to determine the concentration of Cr(VI) in aqueous solution.

The removal capacity (*Q*) is mainly used to characterize the amount of Cr(VI) removal of the adsorbent per gram at equilibrium (mg g^−1^), and it can be calculated by using Eq. (2):

2 $$Q = \frac{{\left( {C_{0} - C_{e} } \right)V}}{m}$$

where *V* is the volume of solution (L), *m* is the mass of PANI/ m-FB composites (g), *C*_0_ and *C*_*e*_ are corresponding to the initial and equilibrium Cr(VI) concentration in aqueous solution (mg L^−1^), respectively.

The removal percentage (*R*) is usually used to characterize the adsorption efficiency, and it can be calculated by Eq. (3):

3 $$R = \frac{{\left( {C_{0} - C_{e} } \right)}}{{C_{0} }} \times 100\%$$

where *C*_0_ and *C*_*e*_ (mg L^−1^) are the concentration of Cr(VI) before and after reaction, respectively.

Figure 1f shows the relation between the contact time and remaining Cr(VI) concentration in the solution after reaction with PANI/m-FB composites. It can be observed that in the beginning stage (0–10 min), the remanent Cr(VI) concentration in aqueous solution declines rapidly, and then the downtrend becomes slowly until equilibrium after reaction for 5 h. As can be seen in Additional file 1: Fig. S2, the Cr(VI) solution becomes colorless and an almost 100% removal percentage was obtained after reaction with composites. This result demonstrated that this kind of composite can effectively remove Cr(VI) in one step.

**Fig. 2:**
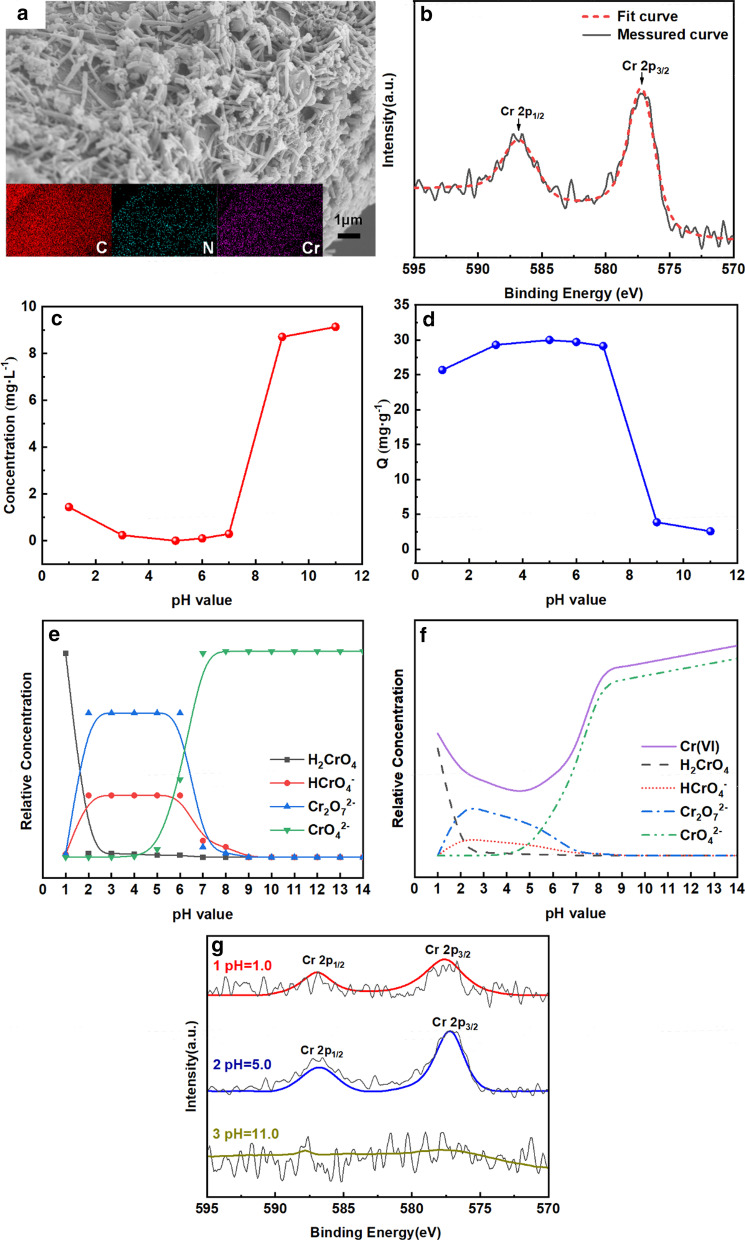
**a** SEM image of PANI/m-FB composites after reaction with Cr(VI) (insert picture: corresponding element mappings); **b** Cr 2p XPS spectrum of PANI/m-FB after reaction with Cr(VI), where Cr 2*p*_1/2_ corresponds to Cr(VI), and Cr 2*p*_3/2_ relates to Cr(III) (*T* = 303 K, *C*_0_ = 10 mg L^−1^, pH = 5.0); effect of pH value on **c** remanent concentration and **d** removal capacity of Cr(VI). (*T* = 303 K, *C*_0_ = 10 mg L^−1^); and the relation of the concentration of different Cr(VI) forms and the pH value of solution; **e** before adding PANI/m-FB composites; and **f** after adding PANI/m-FB composites; **g** Cr 2p XPS spectra of composites after reaction with Cr(VI) solution with different pH values (Curve 1: pH = 1.0; Curve 2: pH = 5.0; Curve 3: pH = 11.0)

Figure 2a shows the SEM image of PANI/m-FB composites after Cr(VI) removal, and it reveals that the morphology of composites changed scarcely compared with the morphology of composites without reaction with Cr(VI) (Fig. 1c). The insert figure in Fig. 2a shows the element mapping of the composites after reaction with Cr(VI), and it can be observed that there exists Cr element besides the inherent elements of composites like C and N. It directly confirms that the lost Cr in aqueous solution has been indeed absorbed by PANI/m-FB composites. What’s more, Fig. 2b shows the XPS spectrum of the PANI/m-FB composites after reaction with Cr(VI) solution. The binding energies locating at 577.3 eV and 588 eV can be assigned to Cr 2*p*_3/2_ and Cr 2*p*_1/2_, respectively, which are corresponding to Cr(III) and Cr(VI) [5, 31, 32]. It was reported that the peak at 577.3 eV can be attributed to Cr(III) by analogy with other chromium compounds [32, 33]. Therefore, it can verify that after treated with the PANI/m-FB composites, Cr(VI) in aqueous solution has been adsorbed on the composites and been reduced to Cr(III) synchronously [31, 34].

To investigate the effect of pH value and initial Cr(VI) concentration on Cr(VI) removal capacity, 1.0 g PANI/m-FB composites were put into 150 mL of Cr(VI) solution with different pH values and concentrations, respectively. Based on the results before, the removal capacity is little difference between reaction for 1 h and 5 h, so we choose the result of reaction for 1 h as the removal capacity.

Figure 2c shows the relationship of residual concentration with the pH values, and it can be seen that when the pH value is below 7.0, the remanent concentration of Cr(VI) in aqueous solution declines until the pH value increases to 5.0 and then increases slightly in the pH range of 5.0 to 7.0. When the pH value is greater than 7.0, the residual concentration of Cr(VI) in aqueous solution increases rapidly. To further investigate the influence of pH value, the removal capacities of PANI/m-FB composites with different pH values are calculated by Eq. (2) and illustrated in Fig. 2d. As we can see, the optimum pH value is 5.0 and the removal capacity is about 29.9 mg g^−1^. Also, in Fig. 2d, it is obvious to see that the removal capacity of composites is stronger in acid condition rather than in alkaline condition.

According to the previous literature, the main existence forms of Cr(VI) in water are chromate (CrO_4_^2−^), dichromate (Cr_2_O_7_^2−^), hydrogen chromate (H_2_CrO_4_ and HCrO_4_^−^), which are dependent on pH values [35, 36]. The balance relationships between these forms are shown as follows [37, 38]:

4 $${\mathrm{H}}_{2} {\mathrm{CrO}}_{4} \, \leftrightarrow \,{\mathrm{HCrO}}_{4}^{ - } + {\mathrm{H}}^{ + } ,\quad {\mathrm{pKa}}_{1} = 0.8$$

5 $$2{\mathrm{HCrO}}_{4}^{ - } \, \leftrightarrow \,{\mathrm{Cr}}_{2} {\mathrm{O}}_{7}^{2 - } + {\mathrm{H}}_{2} {\mathrm{O}},\quad {\mathrm{pKa}}_{2} = - 1.52$$

6 $${\mathrm{HCrO}}_{4}^{ - } \, \leftrightarrow \,{\mathrm{CrO}}_{4}^{2 - } + {\mathrm{H}}^{ + } ,\quad {\mathrm{pKa}}_{3} = 6.5$$

The distribution of different forms of Cr(VI) at different pH can be calculated by Eqs. (4), (5) and (6) using dissociation constant pKa_1_, pKa_2_ and pKa_3_, and the forms distribution diagram of Cr(VI) is illustrated in Fig. 2e. It can be seen that in the solution of strong acid (pH < 2.0), the main existence form of Cr(VI) is H_2_CrO_4_. When pH value ranges from 2.0 to 7.0, the dominant forms of Cr(VI) are HCrO_4_^−^ and Cr_2_O_7_^2−^. And the only stable form of Cr(VI) in alkaline solution (pH > 7.0) is CrO_4_^2−^.

It is important to know that the different forms of Cr(VI) have different oxidation–reduction reaction capacities. According to Eq. (4), in spite that H_2_CrO_4_ is hard to be adsorbed when pH value is below 2.0, some parts of H_2_CrO_4_ could transform to HCrO_4_^−^ in aqueous solution and would be adsorbed and reduced, which can account for the lower removal capacity of PANI/m-FB composites in strong acid solution. When pH value is between 2.0 and 6.8, it is reported that HCrO_4_^−^ and Cr_2_O_7_^2−^ have a higher redox potential, so HCrO_4_^−^ and Cr_2_O_7_^2−^ are easier to be reduced to Cr(III) after PANI/m-FB composite is added. As a result, the removal capacity of PANI/m-FB composite is higher in acid solution. Inversely, CrO_4_^2−^ in alkaline solution is hard to be reduced to Cr(III) due to the lower redox potential [35, 39], and it would cause a lower removal capacity of PANI/m-FB composites as pH value increases. The transformation of Cr(VI) can be seen as follows [5, 16]:

7 $${\mathrm{Cr}}_{2} {\mathrm{O}}_{7}^{2 - } + 6e^{ - } + 14{\mathrm{H}}^{ + } \, \to \,2{\mathrm{Cr}}^{3 + } + 7{\mathrm{H}}_{2} {\mathrm{O}}\quad \left( {E^{0} = 1.33\,{\mathrm{V}}} \right)$$

8 $$2{\mathrm{HCrO}}_{4}^{ - } + 6e^{ - } + 14{\mathrm{H}}^{ + } \, \to \,2{\mathrm{Cr}}^{3 + } + 8{\mathrm{H}}_{2} {\mathrm{O}}\quad \left( {E^{0} = 1.33\,{\mathrm{V}}} \right)$$

9 $${\mathrm{CrO}}_{4}^{2 - } + 3e^{ - } + 4{\mathrm{H}}_{2} {\mathrm{O}}\, \to \,{\mathrm{Cr}}^{3 + } + 8{\mathrm{OH}}^{ - } \quad \left( {E^{0} = - 0.13\,{\mathrm{V}}} \right)$$

Hence, we can infer the change trends of Cr(VI) forms in aqueous solution after reaction with PANI/m-FB composite, just like Fig. 2f shows. In Fig. 2f, the dot lines and dash lines represent the concentration of different forms of Cr(VI) including H_2_CrO_4_, HCrO_4_^−^, Cr_2_O_7_^2−^ and CrO_4_^2−^, respectively, and the solid line represents the total concentration of Cr(VI) in aqueous solution, which is the sum of the other four forms. Notably, the solid line in Fig. 2f has a similar tendency to Fig. 2c.

On the other hand, PANI would be protonated in acid solution and the protonation of PANI is conductive to accelerating the oxidation–reduction reaction for the reason that the doped polyaniline chain with a large amount of positive charges (N^+^) could combine more negative ions HCrO_4_^−^, Cr_2_O_7_^2−^, CrO_4_^2−^ and it contributes to reducing Cr(VI) to Cr(III) [5]. In addition, as negative ions, the main method for Cr(VI) to be adsorbed on the surface of PANI/m-FB is electrostatic interaction. As pH increases, the protonation extent of PANI declines and the concentration of OH^−^ in aqueous solution increases. In consequence, more OH^−^ would compete the adsorption sites of PANI/m-FB with negative ions including HCrO_4_^−^, Cr_2_O_7_^2−^, CrO_4_^2−^ and cause the low removal capacity of Cr(VI) in alkaline condition. Furthermore, the modified fiber ball, which has the large specific surface area and excellent acid resistivity, also contributes to adsorbing heavy metal ions in the acid solution.

Figure 2g exhibits XPS spectra for Cr 2*p* spectra of PANI/m-FB composites after reaction with Cr(VI) at pH = 1.0, 5.0 and 11.0, respectively. In acid condition, two typical Cr 2*p* XPS peaks appeared in Curves 1 and 2 in Fig. 2g, and the peaks at 577.3 eV and 588 eV are corresponding to Cr(III) and Cr(VI), respectively. Comparing Curves 1 and 2, it can be seen that the relative intensity of Cr(III) is higher at pH = 5.0 than that at pH = 1.0, indicating that more reduced Cr(III) was adsorbed in the solution with pH value of 5.0.

It is reported that the dominant form of reduced Cr(III) is gradually changed from Cr^3+^ (pH < 4) to divalent Cr(OH)^2+^ (pH between 4.0 and 4.5) and monovalent Cr(OH)_2_^+^ (pH between 5.5 and 7.0) form as pH increases [11]. That means when pH is below 4.0, the reduced Cr(III) dominantly existed in Cr^3+^ form with 3 positive charges, which is unfavorable to be adsorbed because the increase in electrostatic repulsion between Cr^3+^ and protonated PANI would overcome the chelation interaction between Cr^3+^ and PANI [16]. However, as pH value increases to 5.0, the electrostatic repulsion between the PANI/m-FB composites and Cr(III) is decreased gradually due to the decreased amount of positive charges in the form of Cr(III), and it would cause a little effect on chelation interaction, which is considered as the main factor to adsorb reduced Cr(III) in acid solution. Hence, it could explain the adsorption of reduced Cr(III) is larger when pH value is 5.0 rather than 1.0 in Fig. 2g. Interestingly, in Fig. 2g, as can be seen in Curve 3, there is no obvious Cr 2p peak and this consequence is in accord with the results exhibited in Fig. 2c, d, which further prove that in alkaline solution, the PANI/m-FB composites are scarcely to remove Cr(VI). And based on all of these results, it can summarize that the pH value has a significant influence on Cr(VI) removal and the optimum pH value is 5.0 with the removal capacity of 29.9 mg g^−1^.

**Fig. 3:**
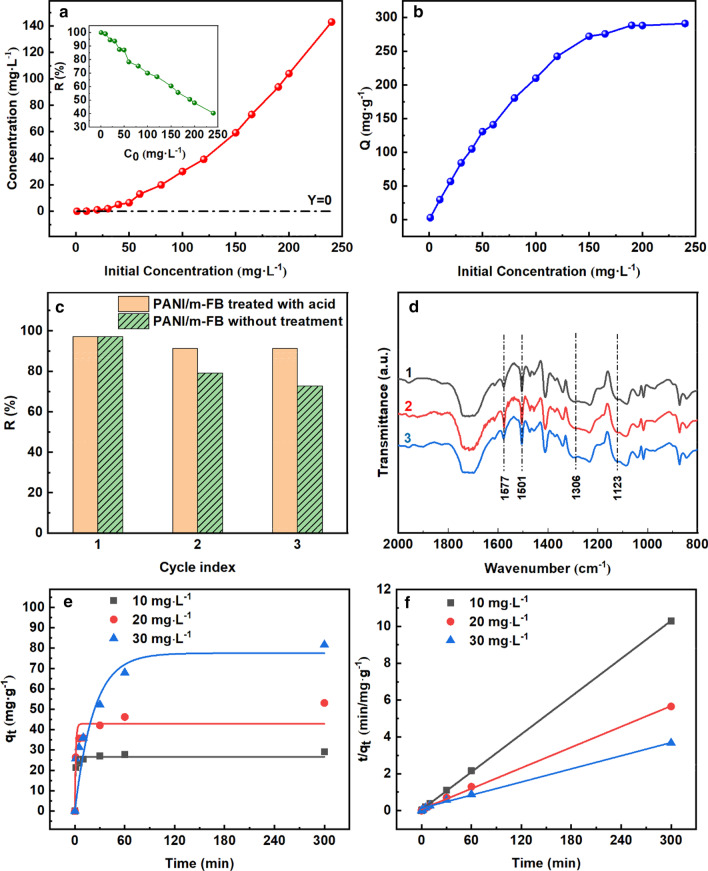
Effect of initial Cr(VI) concentration on **a** remanent concentration (insert picture: the changing of removal percentage); **b** removal capacity of Cr(VI) (*T* = 303 K, pH = 5.0). **c** The removal percentage; **d** the FT-IR spectrum of different composites (*T* = 303 K, *C*_0_ = 10 mg L^−1^, pH = 5.0) (Curve 1: original PANI/m-FB composites; Curve 2: PANI/m-FB composites after treating Cr(VI); Curve 3: PANI/m-FB composites regenerated with acid); **e** pseudo-first-order; **f** pseudo-second-order kinetic model of composites on Cr(VI) removal

Furthermore, initial Cr(VI) concentration in aqueous solution is also an important factor to influence the removal capacity of PANI/m-FB composites apart from pH value. Figure 3a, b illustrates the effect of initial Cr(VI) concentration (*C*_0_) on the remanent concentration and removal capacity, respectively. In Fig. 3(a), it can be observed that as *C*_0_ increases, the residual concentration of Cr(VI) in aqueous solution increases as well. In addition, the dashed line, *Y* = 0, represents a removal percentage of 100% and it shows that the removal percentage is decreased with *C*_0_ increased. The insert picture in Fig. 3a also illustrates that as *C*_0_ increases, the removal percentage obtained by Eq. (3) declines gradually. It can be seen that when *C*_0_ is below 10 mg L^−1^, the removal percentage can reach almost 100%. In order to better understand the effect of initial Cr(VI) concentration on removal performance, the removal capacity is calculated by Eq. (2) and the results are shown in Fig. 3b. We can notice that as *C*_0_ increased, the removal capacity of composites increased as well. When *C*_0_ increases to about 200 mg L^−1^, the removal capacity reaches 291.13 mg g^−1^ and then changes scarcely with *C*_0_ increasing continually. For one thing, this may be due to the limitation of active adsorption sites on the surface of PANI/m-FB composites. In the case of high initial concentration of Cr(VI), there are not enough adsorption sites for PANI/m-FB composites to adsorb and reduce Cr(VI) anymore [16]. For another, due to the high initial concentration of Cr(VI), protonated emeraldine salt has been completely oxidized to pernigraniline base which loses the ability to reduce Cr(VI) [40].

Table 2 exhibits the removal capacities comparison of different adsorbents, and according to this table, it can be concluded that the adsorbent we prepared has a relevant higher removal capacity compared with most of the other adsorbents. Besides, it also has its unique advantages which is easy to be recycled and reused so as to avoid secondary pollution during the process of application.

**Table 2:** The removal capacity comparison with other adsorbents

Adsorbent	*Q* (mg g^−1^)	References
PANI/modified fiber ball	293.13	This work
Activated carbon nanocomposites	500	[41]
Carbon nanotubes	2.517	[42]
Modified zeolites	12.324	[43]
Biosorption	89.32	[44]
Waste weed (*Salvinia cucullata*)	232	[45]
Hollow PANI spheres	127.88	[16]
PANI-coated ethyl cellulose	38.76	[46]
PANI/multiwalled carbon nanotubes	75.59	[47]
Polypyrrole-polyaniline nanofibers	227	[48]
Magnetite/graphene/PANI	153.54	[49]

As we all known, the regeneration and recycling performance is essential for industrialized application. In order to study the regeneration and recycling performance, the used PANI/m-FB composites were taken out and dried for SEM observation and EDS measurement to ascertain the change of composites before and after adsorption. Comparing Figs. 1a and 2c, it can be seen that after reaction with Cr(VI), the morphology of PANI changes scarcely and PANI still coats on the m-FB uniformly and tightly, indicating that PANI/m-FB composites can be cyclic utilization preliminarily.

To further study the regeneration and recycling performances, one group of the used PANI/m-FB composites is regenerated by dispersion in 100 ml H_2_SO_4_ (1 M) for 1 h. For comparison, we did nothing with another group of PANI/m-FB composites except for taking them out and drying. Whereafter, these two groups PANI/m-FB composites were used to deal with Cr(VI) again and the Cr(VI) concentration was determined through the same measure to explore the regeneration and recycling properties of PANI/m-FB composites. Figure 3c exhibits that the removal percentages of PANI/m-FB composites regenerated with acid can still maintain around 90% after repeated for several cycles and are always higher than the composites without acid treatment.

Figure 3d shows the FT-IR spectra of original PANI/m-FB composites (Curve 1), PANI/m-FB composites after treating Cr(VI) (Curve 2) and PANI/m-FB composites regenerated with acid (Curve 3), respectively. Comparing Curve 1 and Curve 2, it can be found that the relative adsorption intensities at 1577 cm^−1^ and 1501 cm^−1^ in Curve 2 are higher than that in Curve 1, suggesting that some emeraldine salt has been oxidized to pernigraniline form during the process of Cr(VI) removal. In Curve 3, the relative adsorption intensities at 1577 cm^−1^ and 1501 cm^−1^ are similar to that in Curve 1, which indicates that the fully oxidized pernigraniline is reduced to emeraldine salt again under the reaction of strong acid.

Therefore, it can be concluded that strong acid can be utilized to reduce the PANI of PANI/m-FB composites from complete oxidation state of pernigraniline to the doped intermediate oxidation state of emeraldine salt. The regeneration of PANI in acid solution is consistent with the reported in the literature previously [50]. Besides, the weights of PANI/m-FB composites weighed shown in Table 1 were measured again after reaction with Cr(VI) and treatment with acid for three times. The results are shown in Table 3, and the PANI load rate of PANI/m-FB composite still remains at the average of 5.2%, indicating that this kind of PANI/m-FB composite has a promising potential to realize industrialized application.

**Table 3:** The PANI load rate of composites after several treatments

	Sample-1	Sample-2	Sample-3
*m*_0_ (g)	4.3030	3.5680	3.6771
*m*_2_ (g)	4.5332	3.7476	3.8694
*η*_2_ (%)	5.34	5.03	5.23

*m*_0_ represents the mass of modified fiber ball; *m*_2_ and *η*_2_ represent the mass of used PANI/m-FB composites and the load rate of PANI after being treated with acid several times, respectively

In order to understand whether the adsorption process is physical or chemical, adsorption kinetics has been studied. For the kinetics experiment, the PANI/m-FB composites (1.0 g) were put into 150 mL Cr (VI) solution with the initial concentration (*C*_0_) of 10 mg L^−1^, 20 mg L^−1^ and 30 mg L^−1^, respectively, and pH of the solution was at 5.0. The kinetic adsorption data were analyzed using two kinetic models: pseudo-first-order and pseudo-second-order kinetic models, respectively. The linear form of pseudo-first-order kinetic model is given by Eq. (10):

10 $$\ln \left( {q_{e} - q_{t} } \right) = \ln q_{e} - k_{1} t $$

And the linear form of pseudo-second-order kinetic model is given by Eq. (11):

11 $$\frac{t}{{q_{t} }} = \frac{1}{{k_{2} q_{e}^{2} }} + \frac{t}{{q_{e} }}$$

where *q*_*e*_ (mg g^−1^) and *q*_*t*_ (mg g^−1^) are the removal capacity at equilibrium time and at random time *t*, respectively, *k*_1_ (min^−1^) and *k*_2_ (g mg^−1^ min^−1^) are the pseudo-first-order and pseudo-second-order rate constants, respectively.

Figure 3e, f exhibits the pseudo-first-order and pseudo-second-order kinetics plot, which are fitted via Eqs. (10) and (11) through the experimental data shown in Additional file 1: Fig. S3, respectively. Notably, by fitting the data with Eqs. (10) and (11), we can obtain the kinetic parameters, shown in Table 4. The pseudo-first-order kinetic model is assuming that the adsorption process is controlled by physical diffusion, while the pseudo-second-order kinetic model supposes that the process is determined by chemical adsorption. From Table 4, it is obvious to see that the pseudo-second-order kinetic model shows a more accurate fit as the relative values of R^2^ are high and the calculated *q*_*e*(cal)_ (equilibrium removal capacity) values are quite close to the experimental Q values (ESI, Additional file 1: Fig. S3(b)). That means the pseudo-second-order kinetic model could give a better explanation to the adsorption process of PANI/m-FB composites. It can be concluded that the procedure may be mainly controlled by chemical adsorption and the rate-limiting step is chemisorption [40, 51].

**Table 4:** Kinetic parameters for adsorption of PANI/m-FB composites

Kinetics models	Parameters	10 mg L^−1^	20 mg L^−1^	30 mg L^−1^
Pseudo-first-order	*q*_*e*(cal)_ (mg g^−1^)	26.591	42.797	77.507
	*Q* (mg g^−1^)	29.161	53.062	81.618
	*R*^2^	0.96293	0.8599	0.95836
	*k* (min^−1^)	1.607	0.091	0.044
Pseudo-second-order	*q*_*e*(cal)_ (mg g^−1^)	29.248	53.533	84.246
	*Q* (mg g^−1^)	29.161	53.062	81.618
	*R*^2^	0.99986	0.99869	0.99586
	*k* (g mg^−1^ min^−1^)	0.0246	0.0045	0.0011

**Scheme 1:**
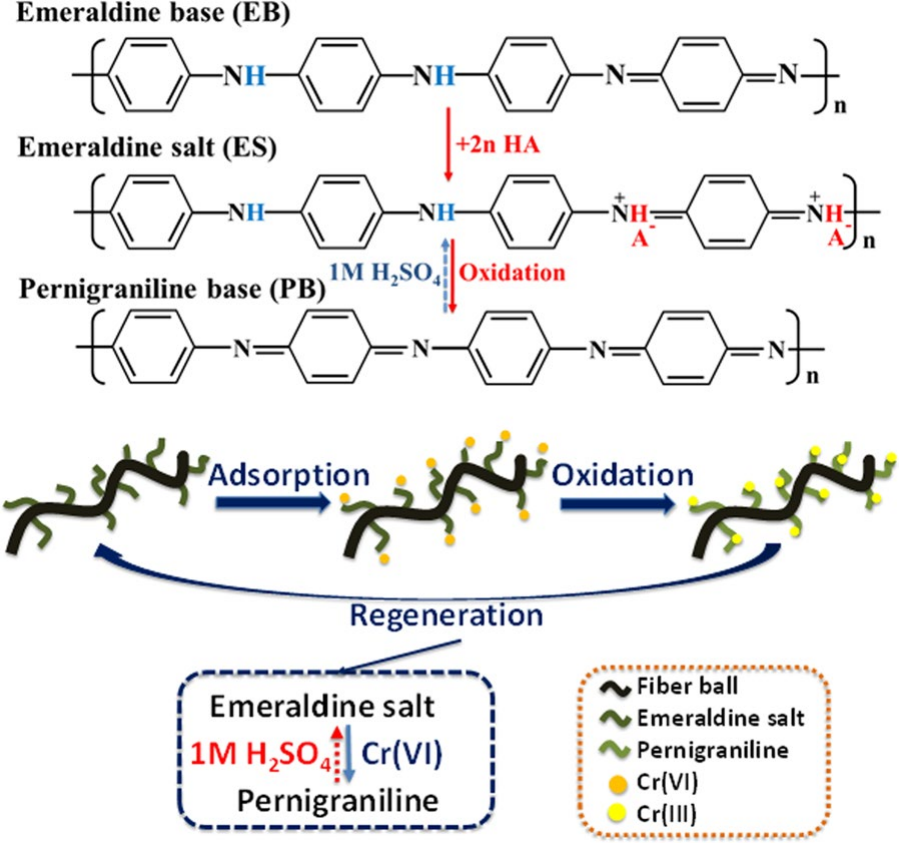
Schematic of PANI/m-FB composites on Cr(VI) removal (A^−^ is counterion)

Based on the above results and analysis, the possible Cr(VI) removal mechanism of PANI/m-FB composites is shown in Scheme 1. After being doped with protonic acid, the synthesized polyaniline in the intermediate oxidation state changes from insulator to conductive emeraldine salt. During the process of Cr(VI) removal, the emeraldine salt of intermediate oxidation state would be oxidized to pernigraniline of the fully oxidized state, and simultaneously, Cr(VI) would be reduced to Cr(III) [11, 13, 52].

Specifically, in Scheme 1, it can be seen that Cr(VI) was first adsorbed on PANI/m-FB composites due to the large number of amine/imine groups of polyaniline, then the adsorbed Cr(VI) was rapidly reduced to Cr(III), and finally the reduced Cr(III) was immediately chelated with PANI and all of these steps of the reaction occurred simultaneously on the same sites of PANI/m-FB composites. Besides, due to the reversibility of the protonated PANI, the fully oxidized pernigraniline can be regenerated into conductive emeraldine salt by means of dealing with strong acid [53].

## Conclusions

In this study, PANI is prepared by means of chemical oxidization polymerization and directly loaded on the modified fiber ball. PANI/m-FB composite with macroscale exhibits an efficient removal capacity of Cr(VI) in aqueous solution. The experiment results show that the maximum removal capacity of the composite is about 291.13 mg g^−1^. And under the condition of *C*_0_ = 10 mg L^−1^, pH = 5.0, the removal percentage is around 100%. Besides, this kind of PANI/m-FB composite not only solves the problem of secondary pollution efficiently due to the macroscale of composites, but also exhibits a well performance of regeneration and multiple utilized after being treated with strong acid. That means PANI/m-FB composites show a promising application in removing Cr(VI) from industrial waste water.

## Supplementary Information

**Additional file 1.** **Fig. S1** (a) Absorbance of Cr (VI) with different concentrations; (b) The fit curve of standard concentration at 350 nm. **Fig. S2** After reaction for 5 h, (a) the photograph; (b) the removal percentage of Cr(VI) solution (C = 10 mg·L , pH = 5.0, V = 150 mL, T = 303 K). **Fig. S3** With different initial Cr(VI) concentrations, (a) the residual concentration in Cr(VI) solution; (b) the removal capacity of PANI/m-FB composite (pH = 5.0, V = 150 mL, T = 303 K).

## Data Availability

The datasets supporting the conclusions of this article are available in the article.

## Abbreviations

APS: Ammonium persulfate
B: Benzene ring
EB: Emeraldine base
ES: Emeraldine salt
EDS: Energy-dispersive spectrometer
FB: Fiber ball
FESEM: Field emission scanning electron microscope
FT-IR: Fourier transform infrared spectroscopy
PANI: Polyaniline
PANI/FB: Polyaniline/fiber ball
PANI/m-FB: Polyaniline/modified fiber ball
PB: Pernigraniline base
Q: Quinone ring
TA: Tartaric acid
UV–Vis: Ultraviolet–visible spectrophotometer
XPS: X-ray photoelectron spectra
