# Supplementary material for: Synthesis of Polyaniline Coating on the Modified Fiber Ball and Application for Cr(VI) Removal
Source: Nanoscale Res Lett. 2021 Apr 8;16:58. doi: 10.1186/s11671-021-03509-y (PMC8032843; doi:10.1186/s11671-021-03509-y)
Supplement: Supplementary file 1 — Additional file 1. Fig. S1 (a) Absorbance of Cr (VI) with different concentrations; (b) The fit curve of standard concentration at 350 nm. Fig. S2 After reaction for 5 h, (a) the photograph; (b) the removal percentage of Cr(VI) solution (C = 10 mg·L , pH = 5.0, V = 150 mL, T = 303 K). Fig. S3 With different initial Cr(VI) concentrations, (a) the residual concentration in Cr(VI) solution; (b) the removal capacity of PANI/m-FB composite (pH = 5.0, V = 150 mL, T = 303 K). [file 11671_2021_3509_MOESM1_ESM.docx]

**Electronic Supply Information**

**Synthesis of polyaniline coating on the modified fiber ball and application for Cr(VI) removal**

Xiao Li Ma ^a, b^, Guang Tao Fei ^a,*^, and Shao Hui Xu ^a^

a. Key Laboratory of Materials Physics and Anhui Key Laboratory of Nanomaterials and Nanotechnology, Institute of Solid State Physics, Hefei Institutes of Physical Science, Chinese Academy of Sciences, P. O. Box 1129, Hefei 230031, P. R. China.

b. University of Science and Technology of China, Hefei 230026, P. R. China.

***Corresponding author**

Guang Tao Fei

Postal address: Institute of Solid State Physics, Chinese Academy of Sciences, P.O. Box 1129, Hefei 230031, P. R. China

Telephone: 86-551-65591453

Fax: 86-551-65591434

Email: gtfei@issp.ac.cn

**
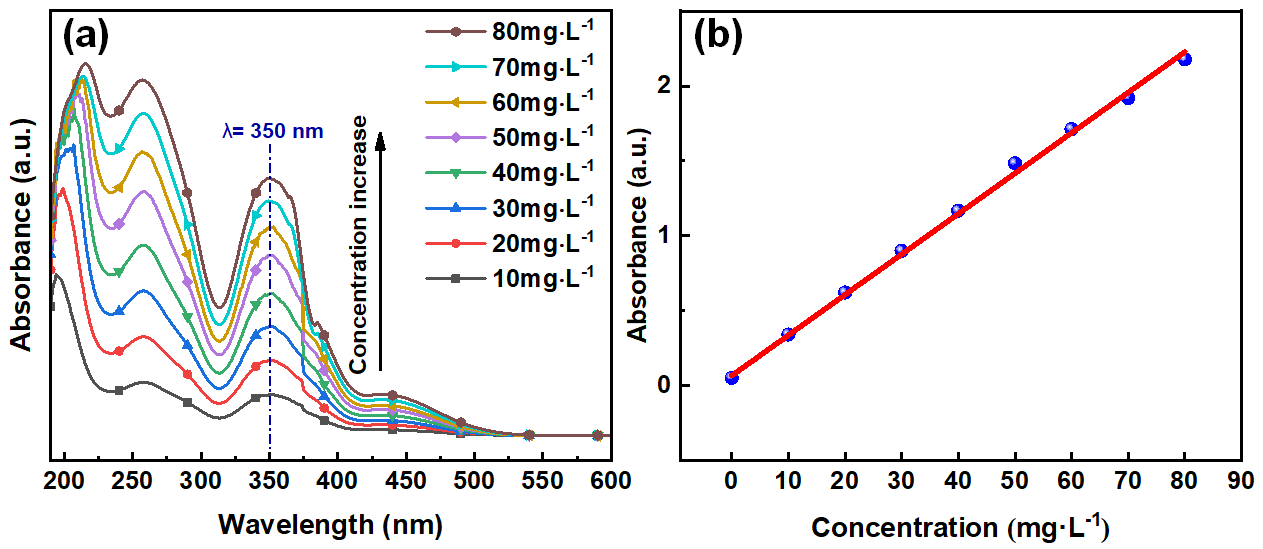
Fig.S1** (a) Absorbance of Cr (VI) with different concentrations; (b) The fit curve of standard concentration at 350 nm

In our experiment, we adopt the method of standard concentration curve to label the concentration of Cr(VI) in solution.


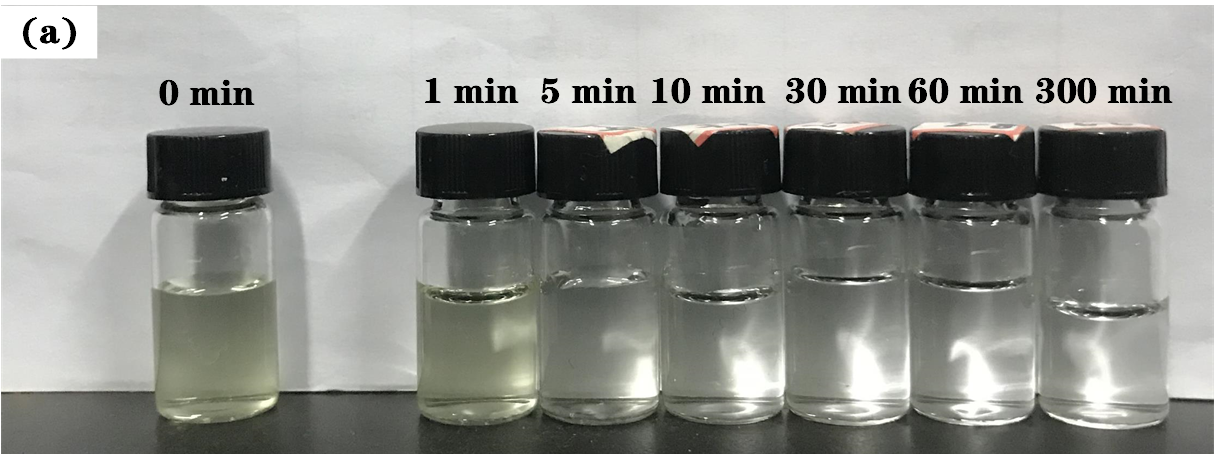


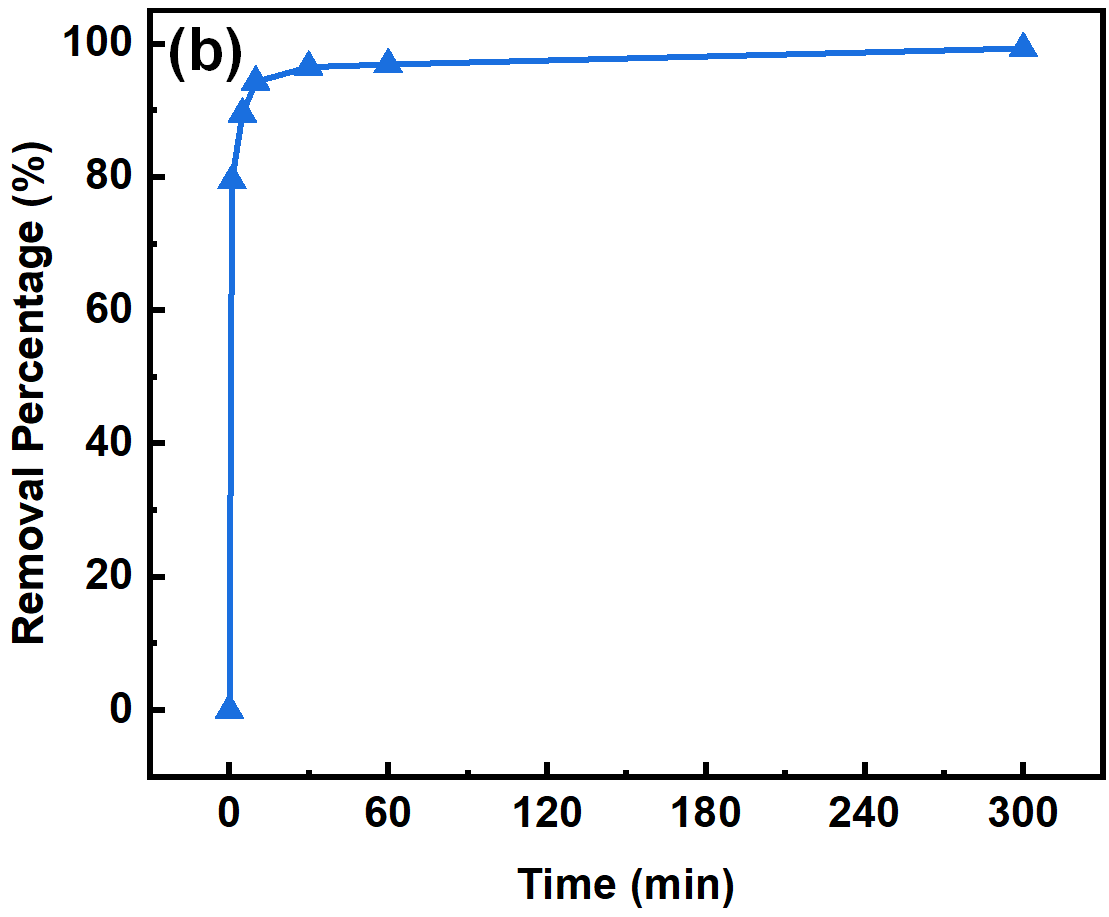


**Fig.S2** After reaction for 5 h, (a) the photograph; (b) the removal percentage of Cr(VI) solution (C_0_=10 mg·L^-1^, pH=5.0, V=150 mL, T=303 K.)

Fig.S2(a) shows the didital images of the wastewater sample after been treated with PANI/m-FB for 5 h and Fig.S1(b) shows the removal percentage of Cr(VI) solution. It can be found that after reaction for 5 h, the removal percentage was up to almost 100% and the flavescens solution became colorless and transparent, indicating that Cr(VI) in the solution has been removed successfully.


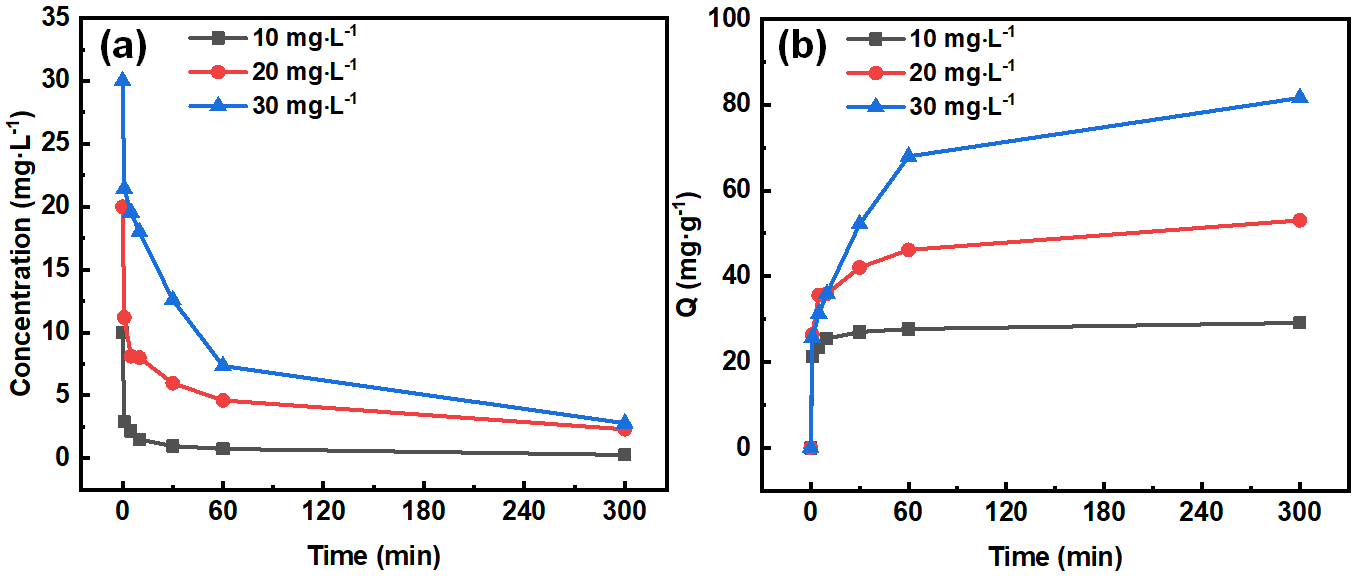


**Fig.S3** With different initial Cr(VI) concentrations,(a) the residual concentration in Cr(VI) solution; (b) the removal capacity of PANI/m-FB composites

(pH=5.0, V=150 mL, T=303 K.)

Fig.S3(a) and (b) exhibit the change of both residual concentration in Cr(VI) solution and the removal capacity of PANI/m-FB composites, respectively, as time goes by with different C_0_.
